# Unsupervised Analysis of Flow Cytometry Data in a Clinical Setting Captures Cell Diversity and Allows Population Discovery

**DOI:** 10.3389/fimmu.2021.633910

**Published:** 2021-04-30

**Authors:** Petra Baumgaertner, Martial Sankar, Fernanda Herrera, Fabrizio Benedetti, David Barras, Anne-Christine Thierry, Denarda Dangaj, Lana E. Kandalaft, George Coukos, Ioannis Xenarios, Nicolas Guex, Alexandre Harari

**Affiliations:** ^1^ Centre of Experimental Therapeutics, Department of Oncology, University Hospital of Lausanne (CHUV), Lausanne, Switzerland; ^2^ Department of Oncology, University Hospital of Lausanne (CHUV), Lausanne, Switzerland; ^3^ Vital-IT, Swiss Institute of Bioinformatics, Lausanne, Switzerland; ^4^ Ludwig Institute for Cancer Research, University of Lausanne (UNIL), Lausanne, Switzerland; ^5^ Bioinformatics Competence Center (BICC), University of Lausanne, Lausanne, Switzerland

**Keywords:** flow cytometry, unsupervised clustering, data-driven analysis, immune monitoring, analytical immunology

## Abstract

Data obtained with cytometry are increasingly complex and their interrogation impacts the type and quality of knowledge gained. Conventional supervised analyses are limited to pre-defined cell populations and do not exploit the full potential of data. Here, in the context of a clinical trial of cancer patients treated with radiotherapy, we performed longitudinal flow cytometry analyses to identify multiple distinct cell populations in circulating whole blood. We cross-compared the results from state-of-the-art recommended supervised analyses with results from MegaClust, a high-performance data-driven clustering algorithm allowing fast and robust identification of cell-type populations. Ten distinct cell populations were accurately identified by supervised analyses, including main T, B, dendritic cell (DC), natural killer (NK) and monocytes subsets. While all ten subsets were also identified with MegaClust, additional cell populations were revealed (e.g. CD4^+^HLA-DR^+^ and NKT-like subsets), and DC profiling was enriched by the assignment of additional subset-specific markers. Comparison between transcriptomic profiles of purified DC populations and publicly available datasets confirmed the accuracy of the unsupervised clustering algorithm and demonstrated its potential to identify rare and scarcely described cell subsets. Our observations show that data-driven analyses of cytometry data significantly enrich the amount and quality of knowledge gained, representing an important step in refining the characterization of immune responses.

## Introduction

The identification of biomarkers and correlates of immune control and protection are key to advance immunotherapy. Flow Cytometry - and more recently Mass Cytometry - have become methods of choice due to their polyvalence, accessibility and throughput. The virtually-unlimited combinations of fluorescent labelled or barcoded antibodies allow rapid profiling of different cell populations or qualitative features in large numbers of samples (1–3). Individual cell subsets including rare cell populations can be distinguished based on combinations of specific markers. Several qualitative cell states can also be captured and those may potentially represent valuable biomarkers that correlate with disease status or clinical outcome (4). During the past decade, technical improvements in instrumentation and reagents led to a massive increase in the volume and complexity of data (5, 6). Consequently, analyses of immunological data obtained by multiparametric cytometry became increasingly complex (7). Not only standard working procedures and instruments need to be increasingly precise, but the complexity of datasets requires specific analytical tools. Distinct analytical strategies were established, in many cases dependent on the nature of the expected outcome. The amount and quality of knowledge gained from these analyses vary and depend on study design and robustness of the workflow.

The application of cytometry is associated with considerable intra- and inter-laboratory variability (8). Harmonization is challenged by the relative lack of golden standards (9). Experts in the field did massive efforts to establish state of the art guidelines (10) to harmonize instruments and operating procedures (11), but also data analysis (12, 13). To increase reproducibility, comparability and accuracy, alternative solutions need to be developed to allow operator-independent, reliable and rapid data processing, including quality-controls and analyses. For years, mathematical algorithms were established and trained to analyse cytometry data in an un-supervised data-driven way, helping researchers to improve the quality of knowledge gained (14). In 2010, the community launched an initiative: Critical Assessment of Population Identification Methods (FlowCAP) to push the development and application of automated tools by organizing tests using yet unpublished raw data made available for research groups to apply their method of choice. Predictions by independent groups were collected to assess how automated methods reproduce state of the art manual analyses. Results were presented and discussed at conferences organized by the FlowCAP consortium and summarized in subsequent articles (15). Since then, numerous additional clustering softwares were developed and their relative performance was reviewed recently (16–18).

In this study, in the context of a prototypic clinical trial, we have performed a comprehensive cross-comparison between two distinct analytical strategies, a state-of-the-art recommended supervised approach (5) and a data-driven unsupervised approach. Longitudinal samples (n=20) from a cohort of five prostate cancer patients under treatment were analysed by flow cytometry to monitor the different cell types identified in peripheral blood (19). Unsupervised analyses were performed in parallel with the clustering algorithm MegaClust, which has been specifically designed to identify clusters in large multidimensional datasets containing various shapes of various densities [https://github.com/sib-swiss/megaclust].

Briefly, MegaClust is a parallel clustering algorithm that takes a matrix of N x D dimensions as input, wherein each row (N) contains the single cell measurements (D) of the various cell surface fluorescent markers studied. The algorithm implements a density-based hierarchical clustering to discover populations (*i.e.* clusters or cell groups). Megaclust regroups two events in the same cluster as soon as their Euclidian distance in the multi-dimensional space is below a certain distance cut-off. Only clusters containing a minimum number of events (nmin) are retained, hence the density-based component. The clustering process is repeated with increasingly larger Euclidian distances allowing existing clusters to grow and new ones to be discovered, hence the hierarchical component. It combines the strength of DBSCAN (20) while capturing at the same time the inherent data structure as OPTICS (21) does. The algorithm has been previously described in detail with performance and robustness benchmarks (22) and successfully used in the past to define immune signature-derived groups of samples resulting in a better stratification of tumours regarding their size compared to manual gating (23).

Our observations from this proof of principle study show that MegaClust captured the complexity of data with more granularity than conventional operator-driven analyses. We believe that the use of mathematical algorithms (e.g. MegaClust) as analysis tool for multi-parametric datasets can help improving data analysis from clinical trials and biomarker identification efforts.

## Material and Methods

### Patient Material and Sample Analysis

We conducted a phase I clinical study (HYPORT, NCT0225474, approved by the Ethical Committee of Canton Vaud and conducted in accordance with the Declaration of Helsinki), in which after signing an informed consent patients with prostate cancer undergoing radiation therapy were treated proposed to donate blood and serum samples for a translational research analysis. Peripheral Blood Mononuclear Cells (PBMCs) from prostate cancer patients were isolated from blood by density gradient using Ficoll-Paque-Plus (GE Healthcare) according to the laboratory standard SOP and immediately cryopreserved in 90% FCS (Fetal Calf Serum) and 10% DMSO (dimethyl sulfoxide) in liquid nitrogen until analysis. Cryopreserved PBMC were thawn in RPMI (Invitrogen), 10% fetal bovine serum (FBS; Gibco), washed and stained with: CD3 APC (BC IM2467, RRID : AB_130788), CD4 PE-Cy7 (BC 737660), CD8 Pacific Blue (BD 558207, RRID : AB_397058), CD14 APC-H7 (BD 641394, RRID : AB_1645725), CD16 FITC (BD 555406,RRID : AB_395806), CD56 PE (BC A07788, RRID : AB_2636814), CD11c Alexa Fluor700 (BD 561352, RRID : AB_10612006), CD19 Brilliant Violet 711 (BD 563036, RRID : AB_2737968), CD123 PerCP-Cy5.5 (eBiosciences 45-1239-42, RRID : AB_10718981), HLA-DR ECD (BC IM3636, RRID : AB_10643231), Zombie UV (77474 Biolegend) as described (5). The samples were directly acquired on a BD Fortessa instrument equipped with the FACS DiVa software (version 8.0.1). Analysis were performed with the FlowJo 9.7.6 (FLOWJO.LLC) and GraphPad Prism v8.

### Megaclust Analyses

For the pilot analysis, the longitudinal data acquired for one patient (patient 0QZW; 4 timepoints) was pooled. After manual gating to remove debris, doublets and dead cells, Megaclust was run at constant events, i.e. selecting the same number of events (25000) in each sample to ensure that each would contribute equally to the cluster discovery phase, whereas remaining events were attributed to the closest discovered cluster in the final phase. Clustering was performed allowing the testing of hierarchical clustering distances ranging from 1 to 500 by increment of 1, or stopping as soon as 95% of events were attributed to clusters, whichever came first. In our case, the latter was true, and unattributed events were reported in a special cluster (cluster 0). To illustrate how the minimal number of events requested to discover a cluster (parameter nmin) impacts the final number of clusters reported, their quality, and their unambiguous attribution to manually gated populations, we ran the algorithm with nmin set to 40 and 120. For the cohort analysis, the 20 samples (4 time points for 5 patients) were pooled and the same process was applied. Since the number of events is 5 times larger than for the pilot study, local clusters densities are increased, and the nmin value requested to create new clusters is reached more easily. Consequently, to obtain a similar number of final clusters as in the pilot study, a nmin value of 80 was chosen. Manually gated reference populations were then used to assign each cluster to a known cell population, when possible.

### RNAseq

Blood from healthy donors were collected from the local transfusion center following the legal Swiss guidelines under the project P_123 with informed consent of the donors and with Ethics Approval from the Canton of Vaud (Lausanne). PBMC from three healthy donors were thawed and directly stained with the aforementioned panel. mDCs (CD123^-^CD11c^+^CD4^+^HLA-DR^+^), pDCs (CD123^+^CD11c^-^CD4^+^HLA-DR^+^) and monocytes (CD14^+^) were sorted and RNA extraction was done with the Promega Maxwell RSC instrument using the Maxwell RSC simply RNA cell extraction kit (Promega AS 1390) following the manufacturer instructions. RNA sequencing was performed by the genomics Technology Facility (GTF) of the University of Lausanne. For the gene set analysis, data were merged with Immgen RNAseq data (GSE122597) for 5 reference profiles containing 83 samples to assess the level of expression of CD4 in the distinct FACS-sorted subsets compared to negative control (B cells, Macrophages and NK) and positive control (CD4 T cells).

## Results

Longitudinal blood samples were collected from five prostate cancer patients treated by radiotherapy (19). In brief, studied patients receive radiation to the prostate at standard doses of 78 Gy in 39 fractions with curative intent. PBMCs were taken isolated at baseline before RT (V1), day 5 of radiation treatment (V2), 15 days after last RT (V3) and 40 to 60 days after the last RT (V4). Peripheral blood mononuclear cells (PBMC) were cryopreserved. All PBMCs (n=20) were thawn, labelled with an antibody panel as described (5) and acquired in one batch to optimize consistency in data acquisition.

### Pilot Analysis: Impact of nmin Parameter

To better illustrate the behaviour of the algorithm upon parameter change, we first compared two clustering runs performed on the same data (pooled samples of patient 0QZW) setting the minimal number of events necessary to detect a cluster (*nmin*) to 40 and 120 (see methods for details). The first run identified 58 clusters, whereas the second run reported 21 clusters. A cell type was assigned to each cluster by comparing their MFI against the manually gated populations. One can observe, for instance, the repartition of CD4^+^ cells into 6 clusters using *nmin* = 120, whereas 15 clusters are reported for CD4^+^ cells using *nmin* = 40 (**Tables 1A**, **B**). Despite this clear overclustering with low value of *nmin*, once regrouped by cell type, these clusters roughly recapitulate the proportion of CD4^+^ events determined by the manual gating analysis (**Table 1A**). We note that using a low *nmin* value is even beneficial in the case of rare populations. For example, with *nmin* = 120, cluster 18 contains a mix of events from mDC and CD4^+^ populations (**Supplementary Figure 1A**), whereas with *nmin* = 40, a clean cluster of mDC cells exempt of CD4^+^ cells is detected (cluster #54, **Supplementary Figure 1B**). Thus, increasing the granularity of the detection by overclustering using low *nmin* values resolves the difficulty to identify rare populations by reporting a large number of clean smaller clusters, which can subsequently be reassigned to their corresponding cell type. This behaviour is further illustrated in **Supplementary Figure 1C**.

**Table 1A:** Table of detected cell numbers and clusters in comparison to MG populations.

population	count.ref	prop.ref	count.pred	prop.pred	clusters n°
**classical**	25528	0.087	25267	0.107	31,32,34,35,49,57
**intermed**	1286	0.004	1197	0.005	46
**non-classical**	1602	0.005	4484	0.019	44,45,50
**Bcell**	13833	0.047	13821	0.059	15,16,21
**CD56bri**	1022	0.003	1492	0.006	37,53
**CD56di**	14426	0.049	15837	0.067	12,13,14,24
**CD8+**	23525	0.081	24460	0.104	10,11,17,18,19,27,29,37,42,43,58
**CD4+**	119640	0.410	113532	0.481	1,2,3,4,5,6,7,8,9,20,22,23,28,38,48
**mDC**	376	0.001	890	0.004	54
**pDC**	820	0.003	3200	0.014	55,56
	**Manual gating**		**MegaClust**	

The number of detected clusters decrease depending on the criteria of minimum cell number per cluster (nmin=40).

**Table 1B:** Table of detected cell numbers and clusters in comparison to MG populations.

population	count.ref	prop.ref	count.pred	prop.pred	clusters n°
**classical**	25528	0.087	24793	0.105	10,11,21
**Bcell**	13833	0.047	14248	0.060	4,5
**CD56di**	14426	0.049	16232	0.069	8,9
**CD8+**	23525	0.081	25208	0.107	6,7
**CD4+**	119640	0.410	112396	0.476	1,2,3,12,13,16
**mDC**	376	0.001	1197	0.005	18
**pDC**	820	0.003	3383	0.014	19,20
	**Manual gating**		**MegaClust**	

The number of detected clusters decrease depending on the criteria of minimum cell number per cluster (nmin=120).

### Conventional Gating Strategy and Cohort Analysis With MegaClust

**Figure 1:**
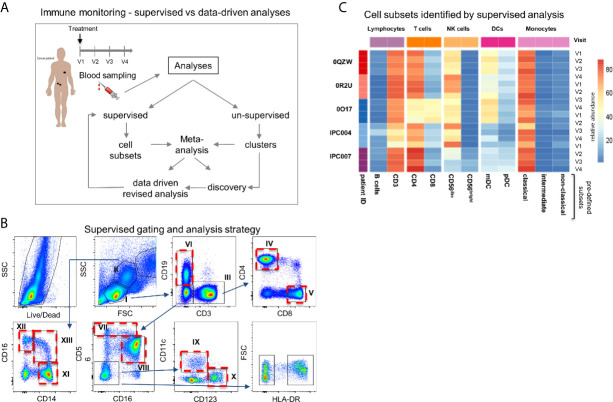
Study design and cross-comparison between supervised and un-supervised data analysis. **(A)** Study design. Longitudinal blood samples were taken from patients initiating treatment. Peripheral blood mononuclear cells were labelled with an antibody panel and FACS data were processed in parallel with a supervised and an un-supervised data analysis. The cross-comparison of data lead to data-driven revised gating strategy and discovery. **(B)** Representative example of the supervised gating and analysis strategy of flow cytometry data. Arrows describe the hierarchical sequences of analysis (*i.e.* gating strategy). Identified cell subsets: lymphocytes (I), T cells (III) (CD3^+^ CD19^-^), CD4^+^ (IV) and CD8^+^ (V) T cells, B cells (VI) (CD19^+^CD3^-^), cytokine secreting NK^bright^ (VII) (CD56^hi^CD16^+^/^-^) and cytotoxic NK^dim^ (VIII) (CD56^dim^CD16^+^) NK cells, mDCs (IX) (CD123^-^CD11c^+^) and pDCs (X) (CD123^+^CD11c^-^); monocytes (II): classical (XI) (CD14^+^CD16^-^), intermediate (XIII) (CD14^+^/^-^CD16^+^/^-^) and non-classical monocytes (XII) (CD14^-^CD16^+^). Ten cell subsets are identified and highlighted with a red dotted frame. **(C)** Heatmap representation of the frequencies of the 10 different predefined cell subsets per patient and time point after supervised data analysis.

**Figure 2:**
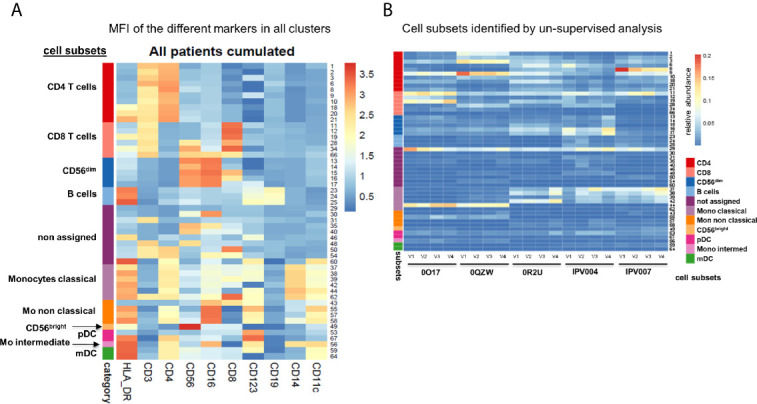
Expression of different markers in all clusters. **(A)** Heatmap of the mean fluorescence intensity (MFI) for each marker in all identified clusters. Cumulative data of all patients and time points (n=20). **(B)** Heatmap representation of 50 good quality clusters identified per patient and time point with the unsupervised analysis using MegaClust. From the 50 clusters, 40 could be attributed to the 10 predefined cell subsets from the supervised analysis while 10 clusters could not be assigned.

The entire dataset was then analysed following a conventional supervised gating strategy and, in parallel, using MegaClust (**Figure 1A**). Using the supervised analysis, 10 distinct cell types were identified (**Figures 1B, C**), including B cells, CD3^+^ T cells divided in CD4 and CD8 T cells, natural killer (NK) cells, monocytes and dendritic cell (DC) subsets. The un-supervised analysis identified 68 clusters, of which 50 were retained as good quality (*i.e.* unimodal distribution). Most of these clusters (*i.e.* 40 out of 50) corresponded to subgroups of the 10 subsets identified in the supervised analysis. However, 10 clusters could not be assigned or associated to any of the supervised subset but did contain a sufficient number of similar cells to be identified as individual clusters (**Figures 2A, B**). We then investigated several examples of discrepancies in the definition of the different cell subsets, including CD4 T cells, CD8 T cells and DCs.

### CD4 T Cell Subsets

**Figure 3:**
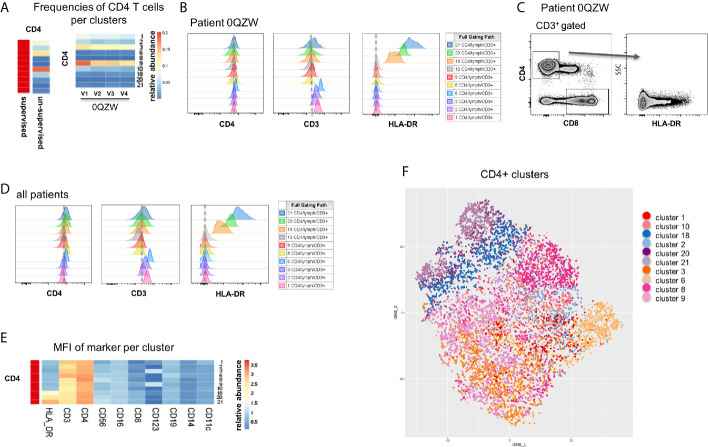
Analysis of CD4^+^ T cell subsets identified by MegaClust. **(A)** Comparison of the different CD4^+^ cell clusters identified by MegaClust. Left panel shows one population of CD4^+^ T cells obtained by supervised analysis, whereas the unsupervised analysis identified 10 clusters within the CD4 T cell population. Four timepoints (visits, V1-V4) are shown for representative patient 0QZW. **(B)** Expression of CD4, CD3 and HLA-DR on the 10 CD4 T cell clusters on V1 from one representative patient 0QZW. **(C)** Expression of HLA-DR on CD4 T cells from patient 0QZW from the supervised dataset. **(D)** Cumulative analysis on all patients and timepoints (n=20) of the expression of CD4, CD3 and HLA-DR on the 10 CD4^+^ cell clusters. **(E)** Cumulative analysis on all patients and timepoints (n=20) of the expression level (mean fluorescence intensity, MFI) of different markers on all CD4 T cell clusters. **(F)** tSNE of all CD4^+^ T cell clusters identified with Megaclust.

One and ten CD4 T cell subsets were identified in the supervised and unsupervised analyses, respectively (**Figure 3A**). By dissecting the ten clusters identified with MegaClust, we noticed that all subsets expressed roughly similar levels of CD4 while striking differences were observed in CD3 expression (**Figure 3B**). Of interest, major differences were observed in HLA-DR expression level, in particular for three of the ten clusters (**Figure 3B**). HLA-DR expression was not taken into consideration in the supervised analyses of CD4 T cells, although a re-analysis confirmed its partial expression on CD4 T cells (**Figure 3C**). This observation was also confirmed by supervised reanalysis with data from all patients together (**Figure 3D**). The differential expression of all markers in these ten clusters indicates that although not all ten clusters are fundamentally unique, CD3 expression levels and, more importantly HLA-DR expression levels, allow the identification of biologically-different subpopulations (**Figure 3E**). Collectively, these observations indicate that distinct CD4 T cell subpopulations were missed with the supervised analysis while those were captured with the unsupervised analysis of MegaClust (**Figure 3F**).

### CD8 T Cell Subsets

**Figure 4:**
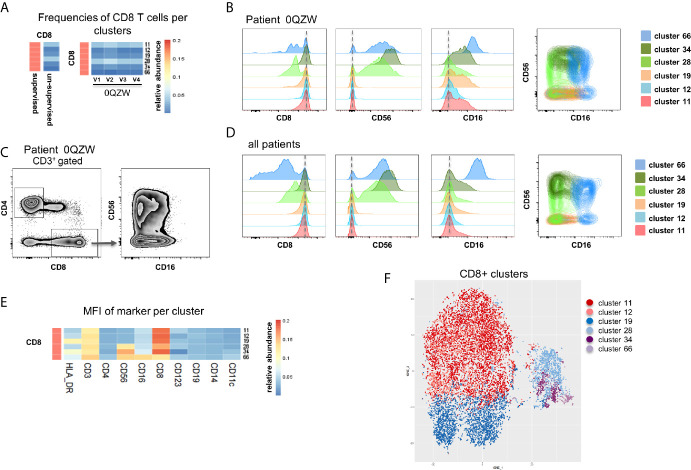
Analysis of CD8^+^ subsets identified by MegaClust. **(A)** Comparison of the different CD8^+^ clusters identified by MegaClust. Left panel shows one population of CD8^+^ cells obtained by supervised analysis, whereas the unsupervised analysis identified 6 clusters within the CD8^+^ population. Four timepoints (visits, V1-V4) are shown for representative patient 0QZW. **(B)** Expression of CD8, CD56 and CD16 on the 6 CD8^+^ clusters on V1 from one representative patient. **(C)** Expression of CD16 and CD56 on CD8^+^ cells on V1 from patient 0QZW from the supervised dataset. **(D)** Cumulative analysis on all patients and timepoints (n=20) of the expression of CD8, CD56 and CD16 on the 6 CD8^+^ clusters. **(E)** Cumulative analysis on all patients and timepoints (n=20) of the expression level (mean fluorescence intensity, MFI) of different markers on all CD8^+^ cell clusters. **(F)** tSNE of all CD8+ cell clusters identified with Megaclust. The NKT-like cell clusters 28, 34.and 66 are clearly separated from the remaining CD8 T cell clusters.

Six CD8 T cell clusters were identified in the unsupervised analysis as compared to one population captured in the supervised analysis (**Figure 4A**). The differences between the clusters were based on a differential expression of CD8, CD56 and CD16 for three out of the six clusters (**Figure 4B**). Re-analysis of cytometry data confirmed the heterogeneous expression of CD56 and CD16 within the CD8 T cell populations, indicating the presence of NKT-like cells (**Figure 4C**). The observation was confirmed by analysing cumulative data collected for all patients (**Figure 4D**). The differential expression of all markers in all six clusters indicates that although not all clusters are fundamentally unique, CD8 expression levels and, more importantly CD16 and CD56 expression levels, allow the identification of biologically-different subpopulations (**Figure 4E** **)**. Collectively, these observations indicate that several real CD8 cell subpopulations were missed with the supervised analysis (e.g. NKT-like) while those were properly captured with the unsupervised analysis of MegaClust (**Figure 4F**).

### mDC and pDC Subsets

**Figure 5:**
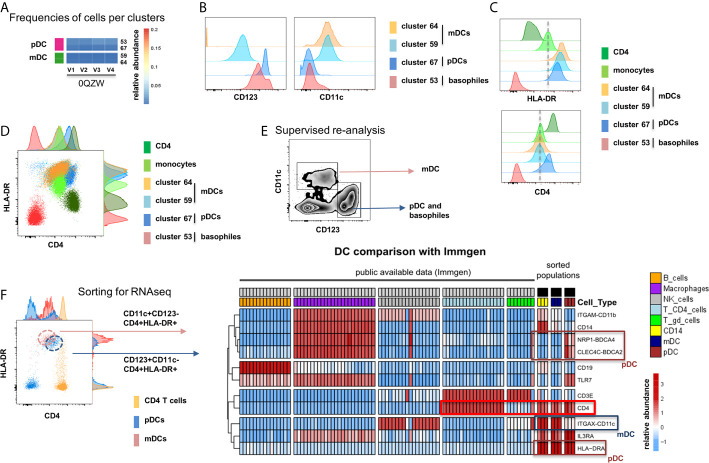
Identification of multiple mDC and pDC subsets by MegaClust. **(A)** Comparison of the different clusters identified by MegaClust as mDCs and pDCs (example for one patient for four time points). **(B)** Expression level of CD123 and CD11c of the two mDC clusters and the two pDC clusters identified by MegaClust. **(C)** Expression levels of HLA-DR and CD4 for the two pDC clusters and the two mDC clusters identified by MegaClust. Cluster 53 could be assigned as basophils due to the lack of expression of CD4 and HLA-DR (25). The expression of CD4 is shown for CD4 T cells and CD14^+^ monocyte clusters for comparison. **(D)** Overlay of CD4 and HLA-DR of MegaClust identified clusters for mDC, pDC, basophils, CD4 and monocytes. **(E)** mDCs and pDCs were discriminated according to CD11c and CD123 expression in the supervised flow cytometry re-analysis according to the new gating strategy described in **Supplementary Figure 5** (one representative patient 0QZW). **(F)** Representative illustration of HLA-DR and CD4 co-expression on mDCs and pDCs prior to FACS sorting CD11c+CD123^-^CD4^+^HLA-DR+ mDCs and CD123^+^CD11c^-^CD4^+^HLA-DR^+^ were FACS sorted (dotted lines). **(G)** Gene expression profiles of flow cytometry sorted CD11c^+^CD123^-^CD4^+^HLA-DR^+^ mDCs, CD123^+^CD11c^-^CD4^+^HLA-DR^+^CD4^+^HLA-DR^+^ pDC and CD14^+^CD16^-^ monocytes from 3 healthy donors are shown in comparison to the RNA expression profile of public available RNAseq datasets (Immgen) indicating the gene expression profile of B cells, macrophages, CD4 T cells, *γ*δ T cells and NK cells.

The unsupervised analysis by MegaClust identified two clusters for mDC (CD11c^+^CD123^-^) and two of pDCs (CD11c^-^CD123^+^) (24) (**Figure 5A**). The different mDC and pDC clusters expressed different levels of CD123 and CD11c (**Figure 5B**) as well as of HLA-DR and CD4 (**Figure 5C**). Collectively, these clusters were also distinct from CD4 T cells and monocytes (**Figure 5C**). One of the cluster assigned to pDC (#53, CD123^+^CD11c^-^CD4^-^HLA-DR^-^) could be identified as basophils given the expression of CD123 and the lack of expression of CD4 and HLA-DR (25) (**Figure 5D**). Supervised re-analysis of the dataset confirmed the expression of CD4 in mDCs and pDCs (**Figure 5E**) which offered the opportunity to purify (FACS sorting) these three populations (*i.e.* HLA-DR^+^CD4^+^ expressing CD11c^+^CD123^-^ mDC, CD123^+^CD11c^-^ pDC and CD14^+^CD16^-^ classical monocytes) from three healthy donors for downstream RNA sequencing analysis (**Figure 5F**). Of interest, the comparison of the transcriptomic profiles of purified populations with publicly available datasets showed that CD4 expression on CD11c^+^CD123^-^ mDC and CD123^+^CD11c^-^ pDC could be confirmed, indicating the accuracy of the unsupervised algorithm used by MegaClust to identify rare and unexpected populations (**Figure 5G**).

Following this observation, we did a meta-analysis of mDCs and HLA-DR^+^CD4^+^ pDCs in PBMCs from prostate cancer (PC) and ovarian cancer (OvCa) patients and frequencies were compared to those identified in healthy donors (HD) (**Supplementary Figure 2**). A significant increase of mDCs in OvCa patients relative to HD was observed.

### tSNE of All Clusters Identified by MegaClust

**Figure 6:**
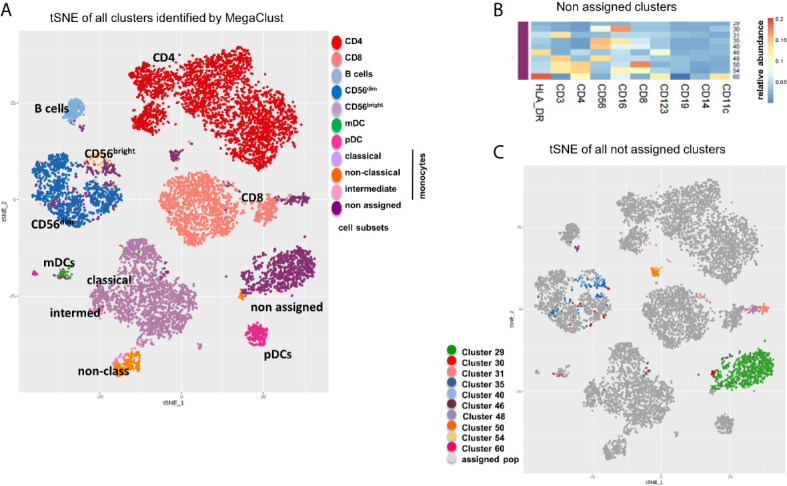
tSNE of all clusters identified by MegaClust. **(A)** tSNE plot showing all clusters and populations identified by MegaClust. The different cell populations are color-coded and listed on the right. One group contains clusters of cells that where not assigned. **(B)** Cumulative analysis on all patients and timepoints (n=20) of the expression level (mean fluorescence intensity, MFI) of different markers on the 10 unassigned clusters. **(C)** tSNE plot showing all clusters and populations identified by MegaClust, with the 10 clusters identified by MegaClust that could not be assigned to known reference populations highlighted with distinct colours.

Finally, a tSNE analysis of all clusters obtained with the unsupervised analysis showed how most subsets were well defined and associated to known immune cell subsets (**Figure 6A**). We also compared the distribution of the populations identified by MegaClust with a tSNE of those identified by manual gating (**Supplementary Figure 3**) and observed a similar distribution, although identified populations are more compact and do not highlight the differences uncovered by MegaClust (*i.e.* inside a given population, groups of cells were less well separated). This could especially be observed in the CD4 T cell and in the NK cell population. Furthermore, by design, manual gating assigns all events in the pre-defined cell populations, and no unassigned clusters were reported. In the unsupervised analysis, 10 clusters could not be assigned to any of those predefined cell populations (**Figure 6C**). These unknown clusters were broadly distributed and expressed heterogeneous levels of common markers (**Figure 6B**), yet they were clearly separated from known cell subsets on a tSNE plot (**Figure 6C**). For example, one of the unassigned cluster (#50) shows a high level of CD3+, CD4+ and CD8+ (**Figure 6B**), and it thus represents CD4+ CD8+ double positive cells, which are well separated from other populations and projected in-between the CD4+ and CD8+ groups in the tSNE map (**Figure 6C**).

By dissecting each cell subset within identified clusters and by analysing these clusters per patient and over time, no major longitudinal trends could be observed, however some fine differences between patients in the relative proportion of events in the various clusters identified for each cell type are apparent (**Supplementary Figure 4**). For example, patient 0Q17 (blue) and patient 0O17 (red) have a high proportion of non-classical monocytes that fell in cluster #55, compared to patients 0R2U (green), IPC004 (grey) and IPC007 (black), that have a high proportion of their non-classical monocytes that fell in cluster #57 and #58 (**Supplementary Figure 4A** left panel). Although these three clusters have been assigned to non-classical monocytes because they have CD14^-^CD16^+^ markers, they exhibit clear differences in their level of expression of other markers (**Figure 2A**). Cluster #55 has a higher level of expression of CD123+ and CD11c+ than other non-classical monocytes clusters indicating a population with DC- or MDSC-like characteristics. The red patient 0O17 also exhibits more differences compared to other patients. For example, this patient has a higher relative proportion of CD8+ T-cells originating from clusters #19 and #34 (**Supplementary Figure 4C** right panel), which bear a high level of HLA_DR marker (**Figure 4E**). Although aggregated counts of the manual gating (**Supplementary Figure 4A** left panel and **D**) also highlight differences for the patient 0O17 (red), the level of details brought by unsupervised analysis is not captured.

Taken together, our observations indicate that supervised analyses have limitations, underestimate the complexity of cellular heterogeneity and hamper discovery. Conversely, unsupervised analyses using mathematical algorithms like MegaClust better capture cell diversity and lead to discovery. These data-driven analyses allowed us to generate an improved and more comprehensive supervised analysis strategy that can be easily implemented (**Supplementary Figure 5**).

## Discussion

Technological advances in the cytometry field drive the inherent needs of novel computational, data-driven approaches, which in turn enable the identification of an unprecedented number of singular cell populations. For a long time, the community has been focused on identifying algorithms that would accurately reproduce results obtained by manual gating and those were considered successful when clustering methods qualitatively and quantitatively identified the same cell populations as the manual gating strategy. This probably has its roots in the way that flow cytometry panels used in clinical setting were carefully designed and validated. These panels are used to quantify the relative amount of specific cell populations determined by the presence of specific surface markers selected based on current knowledge. Marker selection and gating strategy evolved in parallel and predefine cell populations to be profiled. Gating strategies were thus only meant to capture and quantify known cell populations, based on the sequential application of a tree of 1- or 2-dimention gates. In contrast, automated clustering algorithms consider the data as a whole and use all markers simultaneously to assess the similarity between cells. There is a large variety of clustering algorithms with distinct advantages and limitations (26–28), and each algorithm will return different partitions depending on the parameters used. Hence reproducing exactly the same partition as a manually gated analysis (e.g. same number of clusters as target populations) will remain difficult, if not impossible, and may in fact not be desirable. As pointed by Saeys and colleagues (29): “most clustering techniques handle this gracefully by overclustering the data, which assures that all the main structures will be captured, even if they are further split up into smaller populations”. In fact, using unsupervised clustering analysis opens up the possibility to partition the data more finely and can “facilitate the finding of novel and unexpected populations” which can be used to extend and improve gating strategies, as illustrated in this study (16, 29).

Here, we used MegaClust, a hierarchical density-based clustering algorithm where the number of predicted populations depends on the minimum number of events necessary to define a cluster (parameter *nmin*). Although there is some level of subjectivity in the choosing of this parameter, it is easy to understand and can be conceptually linked to the minimal population size that could ever be reported (e.g. setting *nmin* to 1000 on a dataset of 100’000 events will never identify populations less frequent than 1%, and in practice report only much larger populations). From there the number of clusters returned depends only on the inherent data structure, considering all dimensions at the same time. This departs from supervised flow cytometry data analysis, which relies on sequential gating of populations identified visually using pairs of markers, which also comports some level of subjectivity. In that sense, MegaClust, as well as any other clustering algorithms, are unbiased, meaning not biased by human subjective visual perception, since they rely on mathematical computations (*i.e.* distance metric) and can potentially detect undescribed biological populations (e.g., populations in state transition or rare populations) (30). However, the parameter responsible directly or indirectly for the number of clusters (nmin in the case of MegsClust) will have to be fine-tuned, so that the number of clusters reported exceeds the number of biological populations described in the literature. Adequate values are dataset dependent, and our recommendation is to select values which will result in the identification of 50 to 100 clusters. In the subsequent interpretation, identified clusters may be grouped into known cell subpopulations according to common characteristics. These groups of clusters were termed “meta-clusters” (31) or “families” (23). The benefit of overclustering is that it does not preclude the subsequent grouping of clusters to increase the consistency of results compared to manually-gated reference populations (**Table 1A** and **Supplementary Figure 1**) and, at the same time, leaves the possibility to inspect each cluster individually to discover biologically-meaningful subpopulations.

Here, in the context of a clinical trial of prostate cancer patients treated with radiotherapy, we cross-compared state-of-the-art supervised analyses with an unsupervised data-driven analysis. All subsets from the supervised analyses were also captured with MegaClust and their characteristic combination of markers was validated. The MegaClust analysis allowed in addition to distinguish activated from not activated CD4 T cells due to the expression of HLA-DR in three out of ten identified CD4 T cell clusters. Furthermore, a deep inspection of the CD8 clusters clearly confirmed the identification of NKT-like cell subpopulations within the CD8 T cells, a hurdle in supervised analysis based on the various expression level of CD8 during T cell activation. In addition, MegaClust detected the expression of CD4 on HLA-DR^+^ mDC and pDC demonstrating its capacity to identify rare and infrequently reported populations (24, 32).

The overall attempt of this study was to cross-compare supervised and data-driven analysis to determine if unsupervised analysis could bring additional insights, an objective clearly satisfied by MegaClust. The capacity of the algorithm to identify cell subsets considering the expression profile of all markers at the same time departs from the traditional manual gating strategy restricted to the inspection of successive pairs of markers and place MegaClust as a powerful discovery tool that potentially allows discovery of unknown cell populations. The identification of non-targeted (e.g. unknown or not well characterized) low abundance populations in our study is a relevant function in the context of clinical immune monitoring. Although we analysed only a relatively limited cohort of patients, we believe that the use of MegaClust in the discovery analysis phase of clinical trial trials with large cohorts of patients (6, 33) can lead to better interpretation of the data. Our observations indicate that data-driven analyses of cytometry data significantly enrich the amount and quality of knowledge gained which represents an important step in the field of experimental immunotherapies.

Taken together, our study indicates that established supervised (gated) analyses have limitations, underestimate the complexity of cellular heterogeneity and, by design, only capture known populations. Conversely, unsupervised analyses can better capture cell diversity and allow discovery, ultimately leading to improved data-driven supervised analyses able to capture subsets missed in the initial analysis (**Supplementary Figure 5**).

However, one limitation of these methods is that an over-clustering is necessary to capture all populations of interest. Hence, they will generate a number of clusters with very similar characteristics. To limit the effect of over-clustering, it is indispensable to work with datasets acquired under well-standardized conditions and on good calibrated instruments to avoid an impact of batch effect on the clustering. Despite this, the intervention of the researcher will be necessary to decide, which clusters belong to the same cell subset and can be pooled and which clusters belong to a subset of cells with a distinct characteristic expression profile. This step is the frontier between discovery of new cell populations and re-assignment of clusters with only minor differences in the expression of characteristic cell subset markers to already known populations. Although the regrouping of clusters is subject to interpretation, it can be documented and result in the discovery of interesting aspects that would be missed with the conventional gating analysis.

In conclusion, this study demonstrates the added-value of unsupervised algorithms such as MegaClust in the context of biomarker discovery using cytometry data. This is highly relevant to enrich the amount and quality of knowledge gained from discovery efforts and clinical trials, and represents an important step in the field of immune monitoring.

## Data Availability Statement

The datasets presented in this study can be found in online repositories. The names of the repository/repositories and accession number(s) can be found below: https://www.ncbi.nlm.nih.gov/geo/, GSE162177.

## Ethics Statement

The studies involving human participants were reviewed and approved by ClinicalTrials.gov NCT02254746. The patients/participants provided their written informed consent to participate in this study.

## Author Contributions

PB, MS, NG, and AH contributed to conception and writing of the manuscript. PB, MS, FB, DB, and A-CT performed experiments, data acquisition and analysis. FH, LK, and GC provided patient samples. MS, NG, and IX developed MegaClust. PB, NG, DD, LK, GC, and AH contributed to the revision of the manuscript. All authors contributed to the article and approved the submitted version.

## Funding

AH acknowledges support from the Swiss National Science Foundation (310030_182384).

## Conflict of Interest

The authors declare that the research was conducted in the absence of any commercial or financial relationships that could be construed as a potential conflict of interest.

## Abbreviations

DC, dendritic cells; mDC, myeloid DCs; pDC, plasmacytoid DCs; NK, natural killer; MFI, mean fluorescence intensity; MGC, MegaClust.
