# Supplementary material for: Unsupervised Analysis of Flow Cytometry Data in a Clinical Setting Captures Cell Diversity and Allows Population Discovery
Source: Front Immunol. 2021 Apr 30;12:633910. doi: 10.3389/fimmu.2021.633910 (PMC8119773; doi:10.3389/fimmu.2021.633910)
Supplement: Supplementary file 1 [file DataSheet_1.docx]

**Supplementary Figure 1:** **Example of cluster quality evaluation and of the effect of the minimal number of events used to define clusters.** (**A**) Using a large minimal count events to create a cluster (in this case 120) can miss small populations and/or absorb them in other clusters. In this case, the cluster 18 was flagged as poor quality since it presents a bimodal expression profile with one of the mode centered on high level of CD4 and another corresponding to mDC population. Obtaining a large number of bimodal or multimodal clusters in an indication that the minimal number of events to form a cluster should be reduced. (**B**) Using finer clustering parameters, e.g. 40 events sufficient to create a cluster, a clean mDC population (cluster 54) is properly identified. A direct side effect is that large populations are represented by an increased number of clusters. As an example, the manually gated CD4^+^ population (yellow connecting lines) is split in 12 clusters with n=40 instead of only 3 clusters with n=120. (**C**) Comparison of the Megaclust results with the manually gated populations (MGP). The effect of using fine (n=40) or large (n=120) minimal number of events cut-off values is illustrated by lines connecting the clusters obtained with different methods. For example, the manually gated mDC population is correctly retrieved with n=40 (pink connecting line to cluster 54) but is contaminated by CD4^+^ cells when n=120 is used.
