# Supplementary figures and images for: Unsupervised Analysis of Flow Cytometry Data in a Clinical Setting Captures Cell Diversity and Allows Population Discovery
Source: Front Immunol. 2021 Apr 30;12:633910. doi: 10.3389/fimmu.2021.633910 (PMC8119773; doi:10.3389/fimmu.2021.633910)

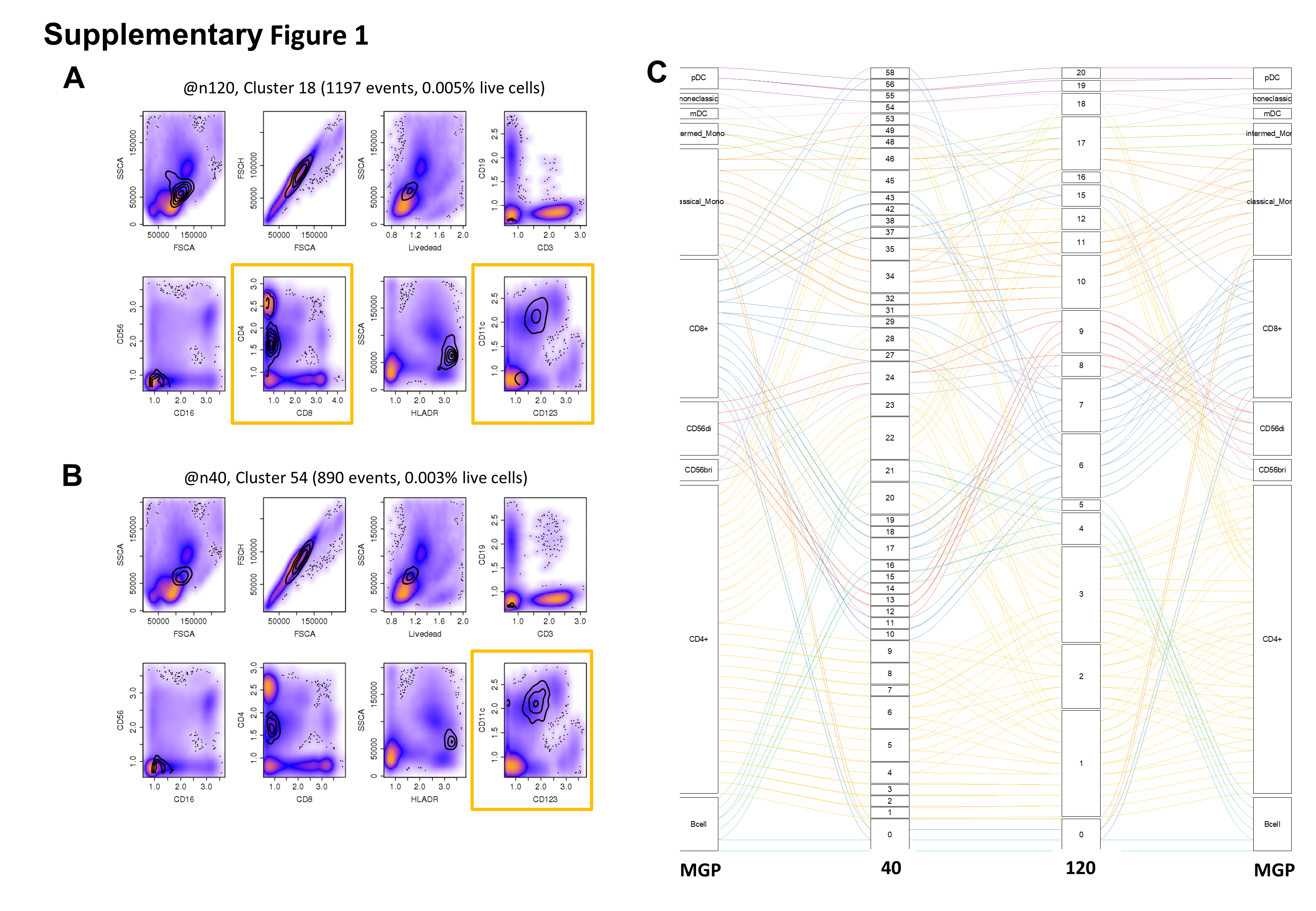

Supplement: Supplementary file 2 [file Image_1.tif]

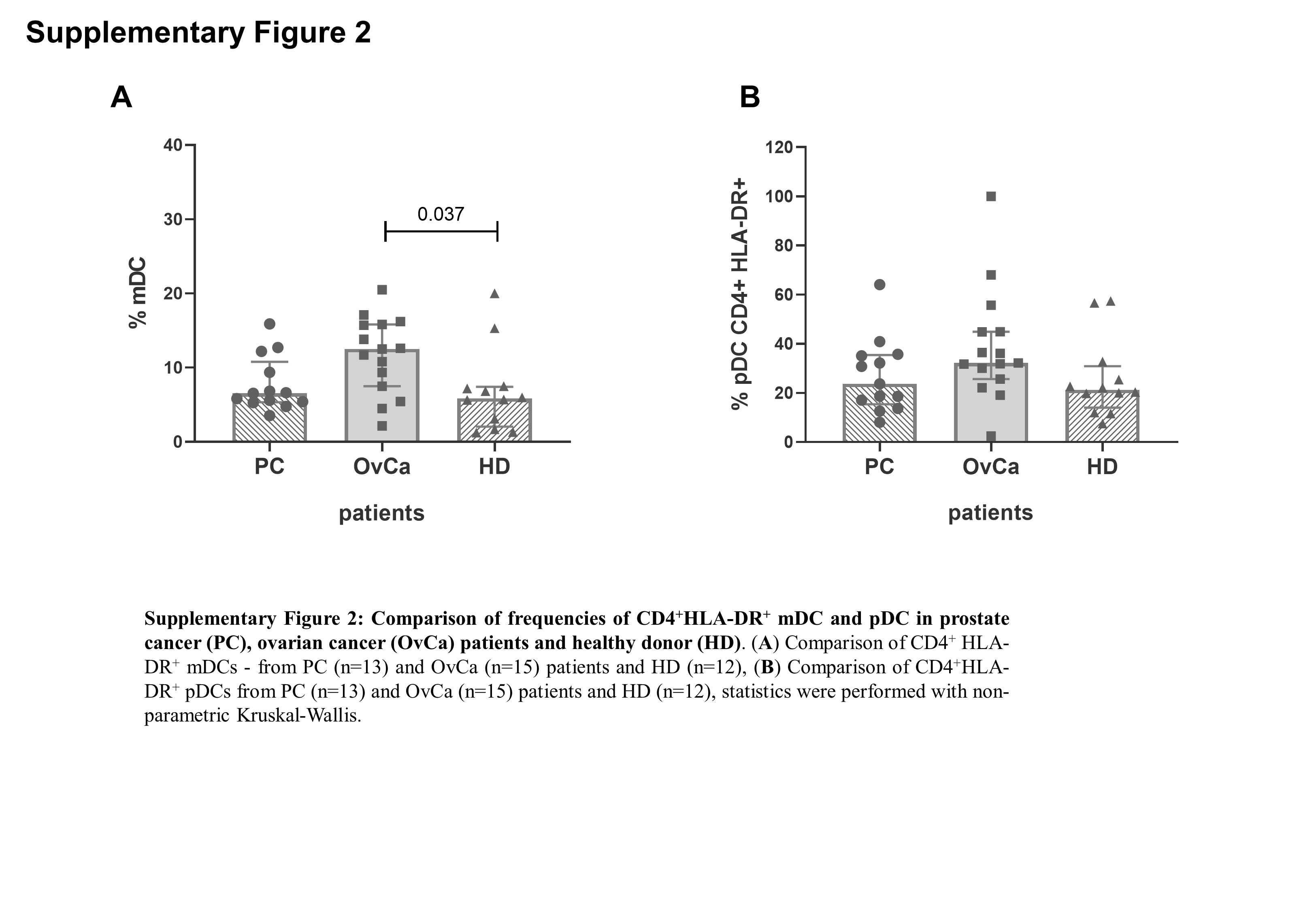

Supplement: Supplementary file 3 [file Image_2.tif]

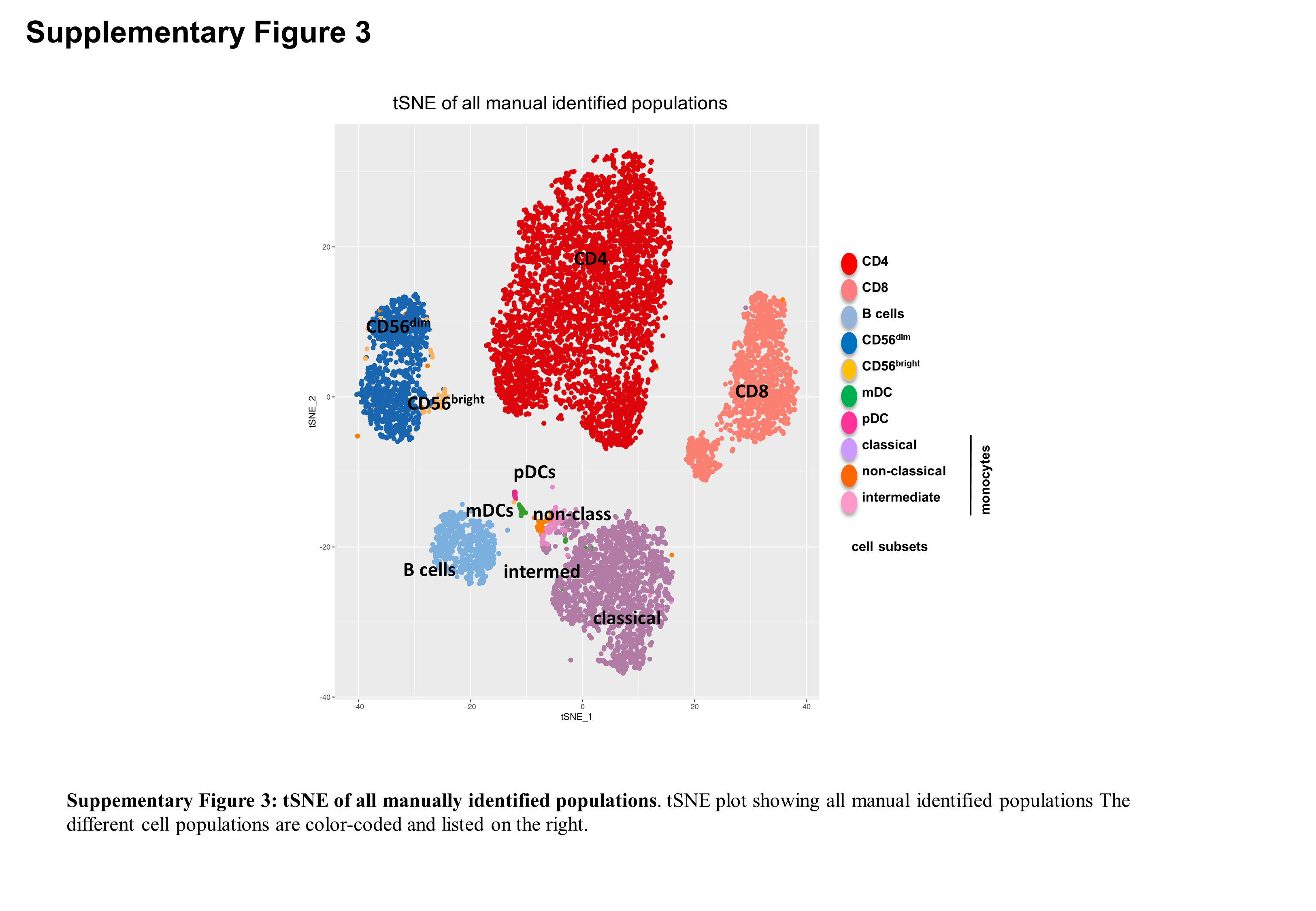

Supplement: Supplementary file 4 [file Image_3.tif]

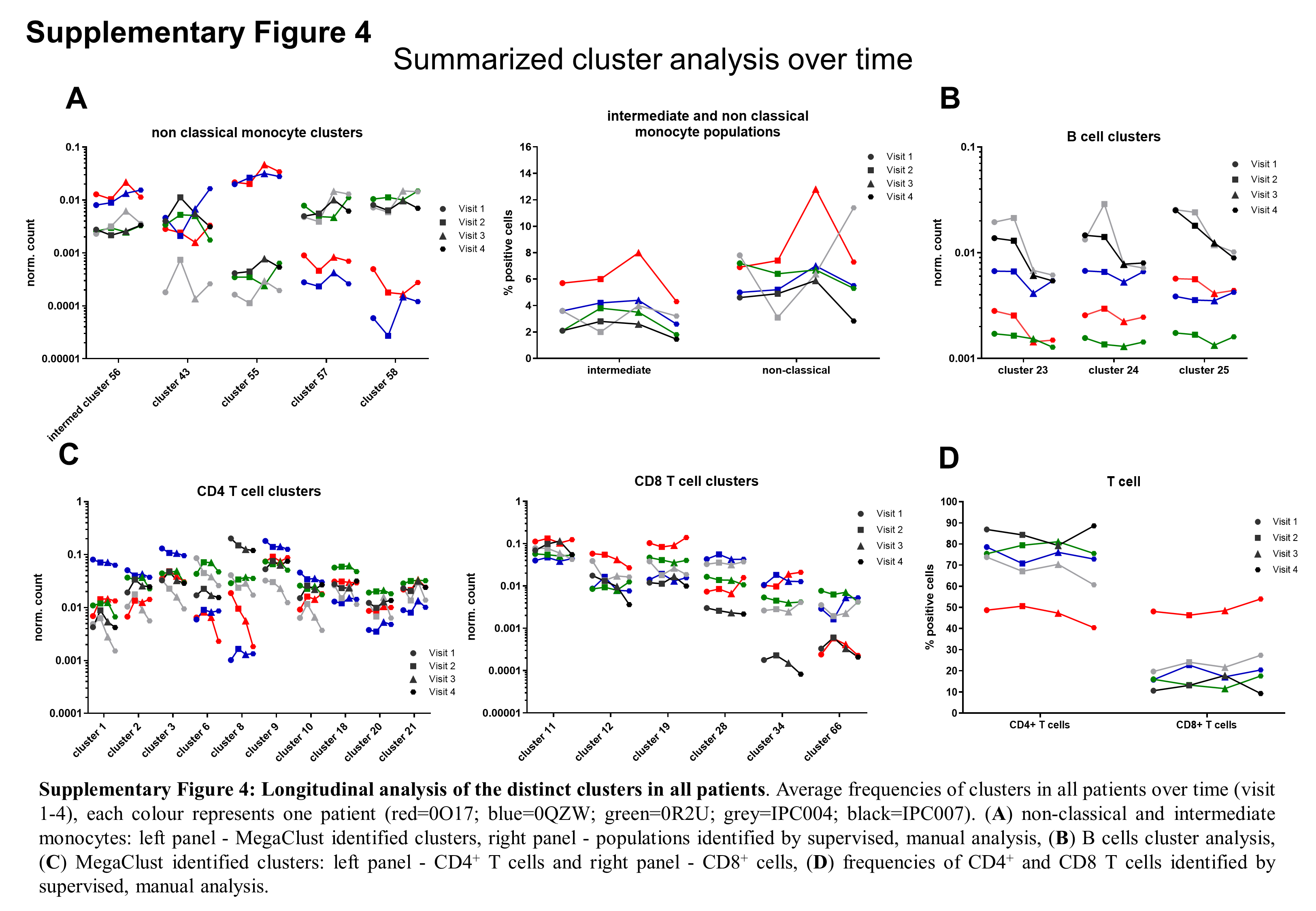

Supplement: Supplementary file 5 [file Image_4.tif]

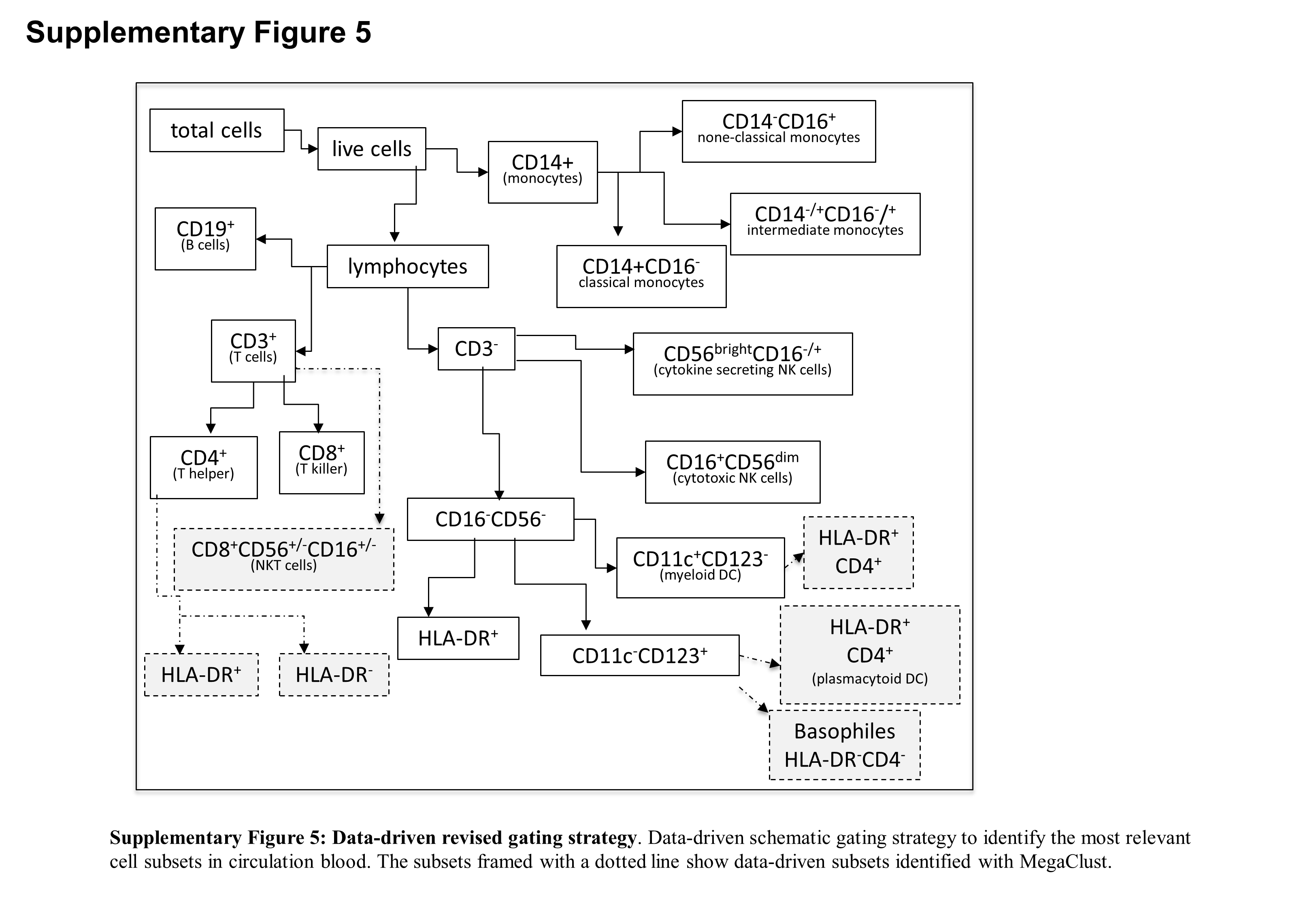

Supplement: Supplementary file 6 [file Image_5.tif]
